# Veterinary fracture diagnosis: a deep learning model for dogs long bone fractures

**DOI:** 10.1038/s41598-026-50387-4

**Published:** 2026-05-14

**Authors:** Ashraf Sobhy Saber, Ibrahim Selim, Heba Askr, Ahmed Elbahgy, Ahmed Ashraf, Ahmed Eldweek, Mostafa Safwat, Mohamed Elsayed, Ahmed AboRashed, Aboul Ella Hassanien

**Affiliations:** 1 Faculty of Vet, University of Sadat City, Sadat City, Egypt; 2 Faculty of Computers and Artificial Intelligence, University of Sadat City, Sadat City, Egypt; 3 Faculty of Computer and AI, Cairo University, Giza, Egypt; 4 Scientific Research School of Egypt (SRSEG), https://egyptscience-srge.com/

**Keywords:** Long bone fractures in dogs, Classification, Deep learning, CNN, Segment anything model, Computational biology and bioinformatics, Engineering, Health care, Mathematics and computing, Medical research

## Abstract

Bone fractures in dogs are common orthopaedic conditions that require accurate diagnosis and rapid intervention. Traditional radiographic interpretation is often time consuming and is subject to variability, emphasizing the need for automated diagnostic tools. This paper represents a deep learning model based on classification of long bone fractures in dogs using medical conventional radiographic images. The proposed model uses a convolutional neural network (CNN), specifically ResNet50 to improve detection and fracture classification. Comparative analysis with other deep learning architectures, including VGG16 and MobileNeTV2, shows the excellent ResNet50 performance. To address the challenge of limited annotated veterinary radiographic datasets, the actual data strategy of augmentation is implemented, which increases the generalization of the model. In addition, the segment model of anything (SAM) is integrated for automated fracture segmentation, allowing precise location and improving diagnostic efficiency. Experimental results show that the ResNet50 achieves high classification performance with an accuracy of 99.76%, accuracy of 99.53%, 100% and F1-score 99.76%, overcoming other competing architecture. These findings emphasize the potential of artificial intelligence in veterinary orthopaedic and offer an efficient, accurate and automated solution for diagnosis and control of long bone fractures in social animals.

## Introduction

Bone or osseous tissue is the hard, semirigid, calcified connective tissue forming the skeleton. Bones are classified as short, flat, irregular, sesamoid and long bones by their shape^1^. Bones are composed of bone tissue, which is sheathed inside and outside by endosteum and periosteum respectively, the bone marrow, as well as blood vessels and nerves^2^.

Long bones are longer than wide, consisting of a diaphysis (body) and two epiphyses (extremities) with their articular cartilage (e.g. humerus, radius, femur, tibia, metacarpals, metatarsals). Long bones are cylindrical and clearly adapted to perform as levers^3^.

Dogs and cats with a variety of orthopaedic conditions are almost seen every day in Veterinary Clinics. If the dog or cat is favouring one leg over another, either continuously or intermittently, there may be a problem with his or her hindlimbs. Sudden limping, difficulty in standing up, slower than normal movement, pain, swelling or if the appearance of the leg itself seems unusual could indicate a broken bone or torn ligament in the dog^4^.

Some dogs and cats require orthopaedic surgery to correct congenital (genetic or hereditary) orthopaedic conditions. Treatment is case-dependent and may include conservative management and/or surgical intervention. Accurate diagnosis of the underlying orthopaedic pathology is essential to select the appropriate management strategy. In traumatic cases, fractures represent one common orthopaedic condition that may require surgical stabilization^5^.

Fractures can be described by anatomic location and joint involvement (extra-articular vs. intra-articular), fracture line configuration (transverse, oblique, or spiral), soft-tissue involvement (closed vs. open), displacement (non-displaced vs. displaced), and degree of fragmentation (simple/two-fragment vs. comminated/multiple fragments; segmental when two distinct fracture lines are present). In addition, Keosengthong et al.^5^ described bone fracture cases in dogs and cats, at a Veterinary Teaching Hospital, Khon Kaen University, Thailand (2013–2016), classified on type as: Transverse, Comminated, Oblique, Physeal, Spiral, Fissure, Condylar, Compression, Avulsion, Green stick, where fresh fractures are classified based on the number of fragments, the type of bone, the fracture site within the bone, and whether they are open or closed and whether the fracture is complete or incomplete^6,7^.

Artificial Intelligence (AI) has shown significant promise in the field of veterinary medicine, particularly in diagnosing long bone fractures in dogs and cats through the analysis of medical images. By utilizing advanced machine learning techniques, AI systems can analyze radiographs (Conventional radiographic images) to identify fractures that may be subtle or easily overlooked by veterinary practitioners. The process typically involves training AI models on large datasets of annotated images, where fractures have been previously identified by veterinary professionals. Once trained, these models can assist veterinarians by providing a second opinion, highlighting potential fractures, and even classifying the type and severity of the injury. This can lead to faster diagnosis and treatment, ultimately improving patient outcomes^8^.

Moreover, AI can enhance the efficiency of veterinary practices by reducing the time spent on image analysis, allowing veterinarians to focus more on patient care. As technology continues to evolve, the integration of AI in veterinary diagnostics is expected to become more prevalent, offering a valuable tool in the assessment of musculoskeletal injuries in companion animals^8^.

In recent years, Deep Learning (DL) algorithms, including health care and biology, have been used in various areas^9–11^. The DL architecture, in particular a convolutional neural network, serves as strong elements for the tasks of image-based, including detection of fractures, tissue and organs’ segmentation and disease identification. Identification of fractures in human bones has proven to be an important subject of interest. However, due to lack of investigation on this topic within veterinary medicine, there is a gap in literature. This research was carried out to strengthen existing literature in veterinary medicine. Using the capabilities of DL, veterinary medicine can increase diagnostic accuracy, reduce expenditures and increase the quality of treatment for animals. Research in this area is continuous and represents a significant potential for the future of veterinary diagnostics. The primary contributions of the proposed model in this paper are as follows:

1. Using the power of DL in diagnosis the long bone fractures in dogs addressing a gap in this field.
2. The proposed ResNet50 model achieves good accuracy compared to other competing algorithms such as VGG16 and MobileNetV2.
3. Developing a custom model for data augmentation to address the limitation of available images, enhancing the model’s generalization ability, accelerating diagnosis, and improving accuracy.
4. Implementing the Segment Anything Model (SAM) for automated fracture segmentation, enabling precise localization of fractures and improving the overall diagnostic process.

The structure of this paper is organized as follows. Section  2 provides a literature review and discusses previous work in the field. Section  3 outlines the research methodology and framework of the proposed model. Section  4 focuses on the implementation details, results, and discussions. Finally, Sect.  5 concludes the paper and discusses future directions for research.

## Related work

Despite the promising advancements in DL for classifying long bone fractures in dogs, there remains a significant gap in the literature, particularly in the application of DL models tailored specifically for veterinary radiology. The existing studies, while demonstrating high classification accuracy using CNNs, RNNs (recurrent neural networks) and transfer learning models, often suffer from limitations such as small or imbalanced datasets, lack of external validation, and challenges in model interpretability. This section presents the literature review of the current research in the filed ending with a summary report of the limitations as in Table 1.

Al-Shara et al.^12^ developed an automatic system to detect long bone fractures using clinical Conventional radiographic images. Diagnosing bone fractures is a critical and time-consuming task, and accurate identification of fractures can save time and reduce errors. To improve diagnosis, feature extraction is performed using Histogram Oriented Gradients (HOG) and Local Binary Pattern (LBP). The study employs two classifiers: Support Vector Machine (SVM) and Multilayer Perceptron (MLP). While the SVM classifier achieves 97.85% accuracy using the Radial Basis Function (RBF) kernel, MLP achieves a higher accuracy of 99.15%, with perfect sensitivity (100%) and a specificity of 98.35%. The study demonstrates a computer-based long bone fracture detection system using MATLAB, providing valuable insights and offering potential as a tool for improving fracture diagnosis in clinical settings.

Ergün et al.^13^ aimed to determine the maturity of dogs, date fractures, and detect long bone fractures using deep neural networks like AlexNet, ResNet50, and GoogLeNet. The performance of these sub-studies is evaluated using accuracy and F1 score. ResNet-50, AlexNet, and GoogLeNet are the most successful algorithms for the three tasks, with F1 scores of 0.75, 0.80, and 0.88, respectively. The study could be developed into support tools for practicing veterinarians to improve the treatment of dogs with fractured bones.

Hauback et al.^14^ presented a convolutional neural network (CNN) model to diagnose and assess the severity of elbow dysplasia in dogs using Conventional radiographic images. With 7229 images collected from clinics in Norway, various EfficientNet models were tested for classification. The highest-performing model achieved a 95.8% test accuracy for four-class classification. Additionally, image pre-processing methods and explainability analysis were conducted, revealing limitations in model reliability, especially for clinical applications. Although not a fracture condition, we include this work briefly as a comparative example of DL applied to conventional radiographic images in veterinary practice, highlighting common issues of validation and interpretability.

Baydan et al.^15^ aims to classify tibia fractures and localize fracture sites in digital images of cats and dogs using deep learning. A dataset of 1,488 images was used, collected from universities and institutions. Three approaches were implemented. In the first study, tibia fractures were classified using Mask RNN (accuracy: 74%, F1 score: 85%), followed by fracture localization (F1 score: 84.5%). The second study directly localized fractures using Mask R-CNN (accuracy: 52.1%, F1 score: 68.5%). The third study applied SSD Single Shot Multi Box Detector (SSD) for localization (F1 score (the harmonic mean of precision and recall): 46.2%).

Ali et al.^16^ uses ML to introduce a multi-class classification and detection system for long bone fractures. This study uses binary and multi-class image classifications and an image detection model to distinguish normal and fractured bone Conventional radiographic images. Models A, B, and ResNet50 are used for grayscale images, while a ResNet50 fine-tuned model and a Faster RNN detection model is used for fracture type identification. The accuracy rates are high.

Alshaharn et al.^17^ develop an effective system of bone fracture classification using deep learning models, such as Yolov8 and VGG16 to detect and classify bone fractures from X -ray images. The models were evaluated in terms of their accuracy in fracture diagnostics, while Yolov8 achieved an accuracy of 73.4%, which represents its strength to detect an object in real time, while VGG16, popular CNN, was also used for classification tasks. The use of the tuning of the hyperparameter and data augmentation significantly improved the performance of the model. These findings emphasize the potential of deep learning algorithms in helping doctors with a more accurate and efficient fracture diagnosis, which eventually improves the patient’s results.

Alkhatib et al.^18^ emphasize the potential of RNN and CNN to detect bone fractures during medical imaging. RNN shows promising due to its high accuracy and robustness across various optimization techniques (Adam and SGD). However, the authors emphasize the need for further research of tuning hyperparameters, diversity of data sets and external validation to ensure the reliability of models in clinical practice. The contribution shows that deep learning models, especially CNN and transmission approaches, are highly effective for the classification of bone fractures in X -ray images. Models achieve high accuracy (up to 95%) and can serve as a reliable baseline for future research. However, the authors emphasize the need for larger data sets, better interpretability and verification in the real world to make these models practical for clinical use.

**Table 1:** A summary of the current literature for AI models in identifying of long bone fractures in Dogs.

Ref.	Year	Model	Performance	Limitations
^12^	2022	SVM (RBF Kernel), MLP	SVM: 97.85% accuracy, MLP: 99.15% accuracy	Small dataset, lack of generalizability. The model’s performance may be limited by dataset quality, hyperparameter tuning methods, and the lack of detailed patient history or fracture types. Further work is needed to improve generalizability and optimize preprocessing steps.
^13^	2021	ResNet50, AlexNet, and GoogLeNet	The F1 scores: AlexNet: 0.75, ResNet50: 0.80, GoogLeNet: 0.88.	The model’s performance is relatively small, and further work is needed to develop comprehensive support tools for veterinarians.
^15^	2021	R-CNN, SSD	R-CNN: Accuracy: 74%, F1 Score: 85%.	Potential for improvement in accuracy; Limited to images classified in Phase 1; Lower accuracy compared to the first study; Lowest performance among the three approaches
^16^	2024	ResNet50, Faster RNN,	ResNet50 with Binary Classification = 96.5% , ResNet50 Multi-class Classification = 87.7% ,	The models’ detection capabilities (especially Faster RNN) are limited to fracture location identification. Potential issues may arise from dataset variability and image quality.
^17^	2024	DL model: YOLOv8 and VGG16.	F1 score : YOLOv8 = 80%, VGG16 = 72.22%	Data augmentation can improve performance but cannot fully capture the complexity of Conventional radiographic images. Both YOLOv8 and VGG16 are DL models with limited interpretability.
^18^	2021	RCNN, CNN	F1 score: RNN = up to 99%, CNN = up to 97%	The study focuses on binary classification of fractured vs. healthy bones, but clinical fractures vary in type and severity, requiring more detailed classification.

## The proposed model

**Fig. 1:**
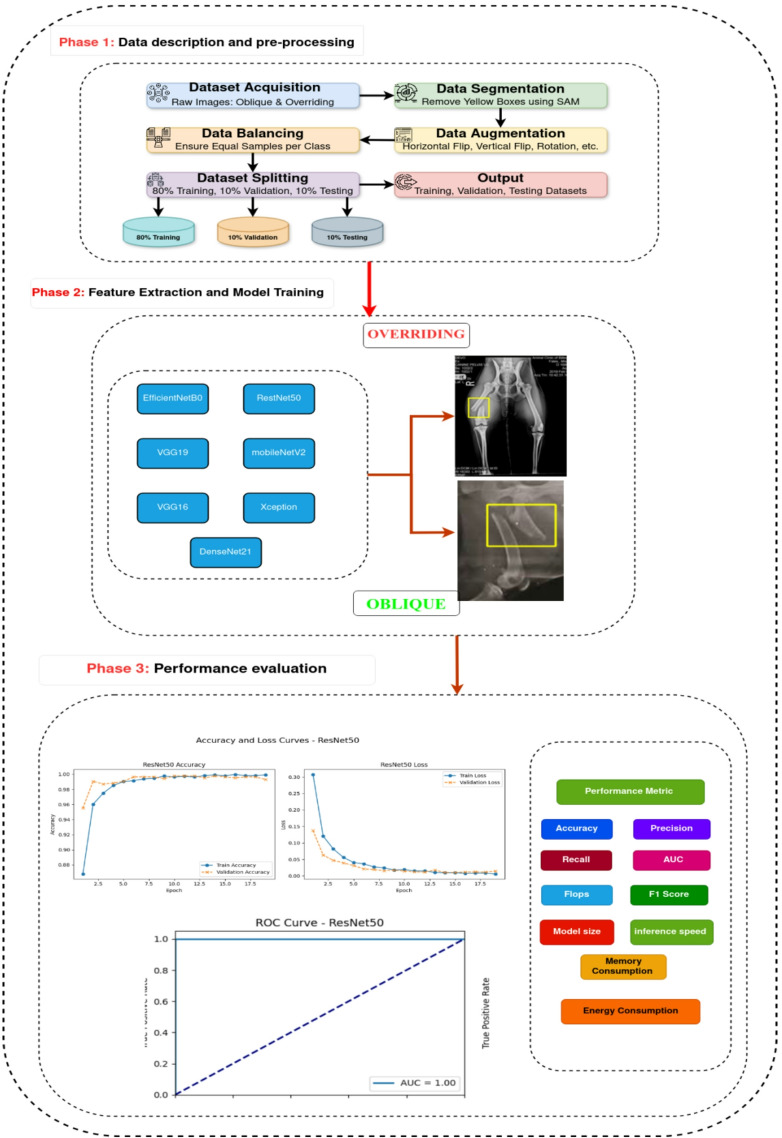
The proposed model.

This research presents proposed model for classification of dog’s bones fractures using multiple pretrained models like ResNet50, VGG16, VGG19, EfficientNetB0, Xception, MobileNetV2 and DesNet121. The proposed model consists of three phases as presented in Fig. 1, and these phases will be illustrated in detail in the following sections.

### Phase 1: data description and pre-processing

**Fig. 2:**
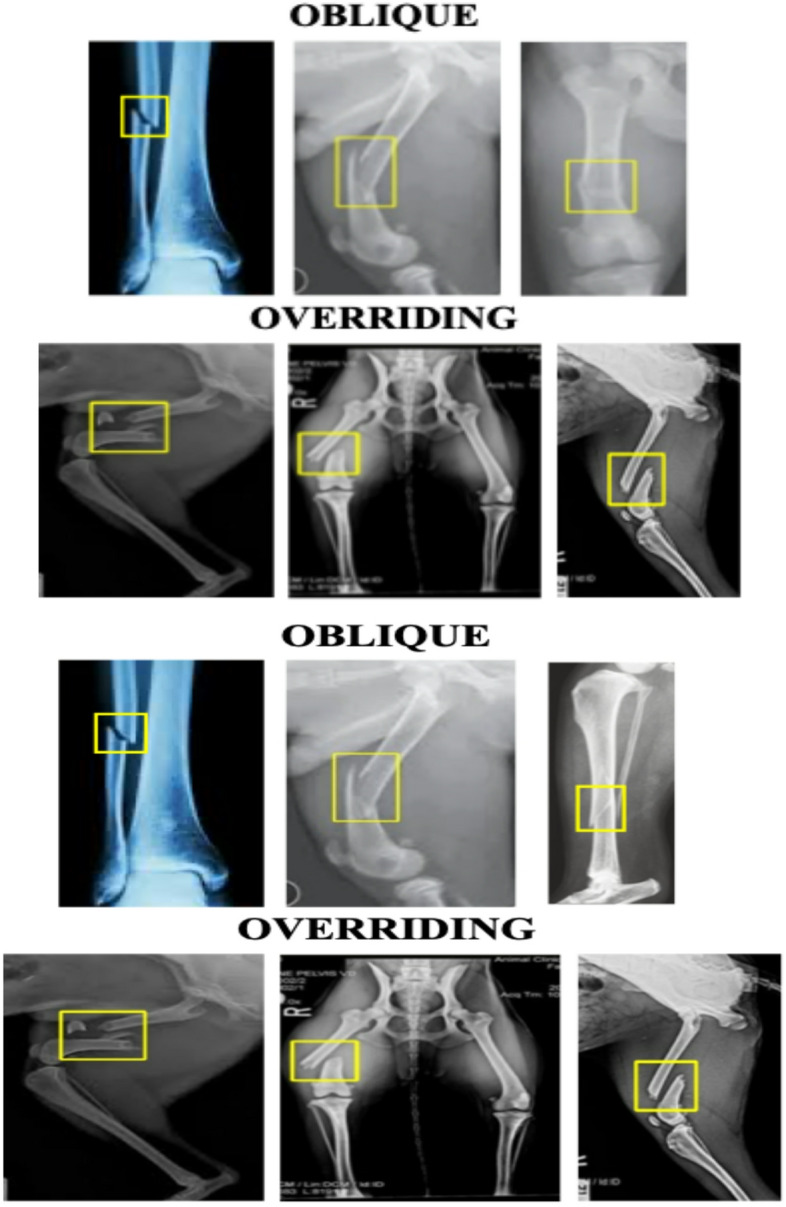
Samples selected from the dog long bone fractures dataset.

The dog long bone fractures image dataset consists of 44 images (15 for oblique, and 29 for overriding), self-collected from different source such as Lawndale Veterinary Hospital website, and Animal Clinic of Billings website in addition to the Wikipedia (images of dogs long bones fractures)^19–21^. Figure 2 presents samples from this dataset.

#### Data segmentation

To ensure optimum input quality for the following classification phase, we have implemented a strict step of pre -processing on data segmentation. Specifically, we focused on the area inside the yellow box present in the data files, as this area consistently contained a dog fracture-prime area of ​​interest for our study. By segmenting and insulation of this area, we tried to increase the performance of the proposed DL by directing its attention to the most relevant anatomical features and minimizing the influence of irrelevant basic information. To this end, we used the Segment Anything Model (SAM), the most modern segmentation algorithm developed by^21^, which allowed the precise definition of the fracture in the yellow box and improved the accuracy and generalization of our model.

The SAM algorithm accurately identified yellow boxes by creating segmentation masks, separating features from relevant image information. This strategy improved data clarity and focused the classification model on significant functions during training, reducing the risk of unsettling correlations or distortions.

**Fig. 3:**
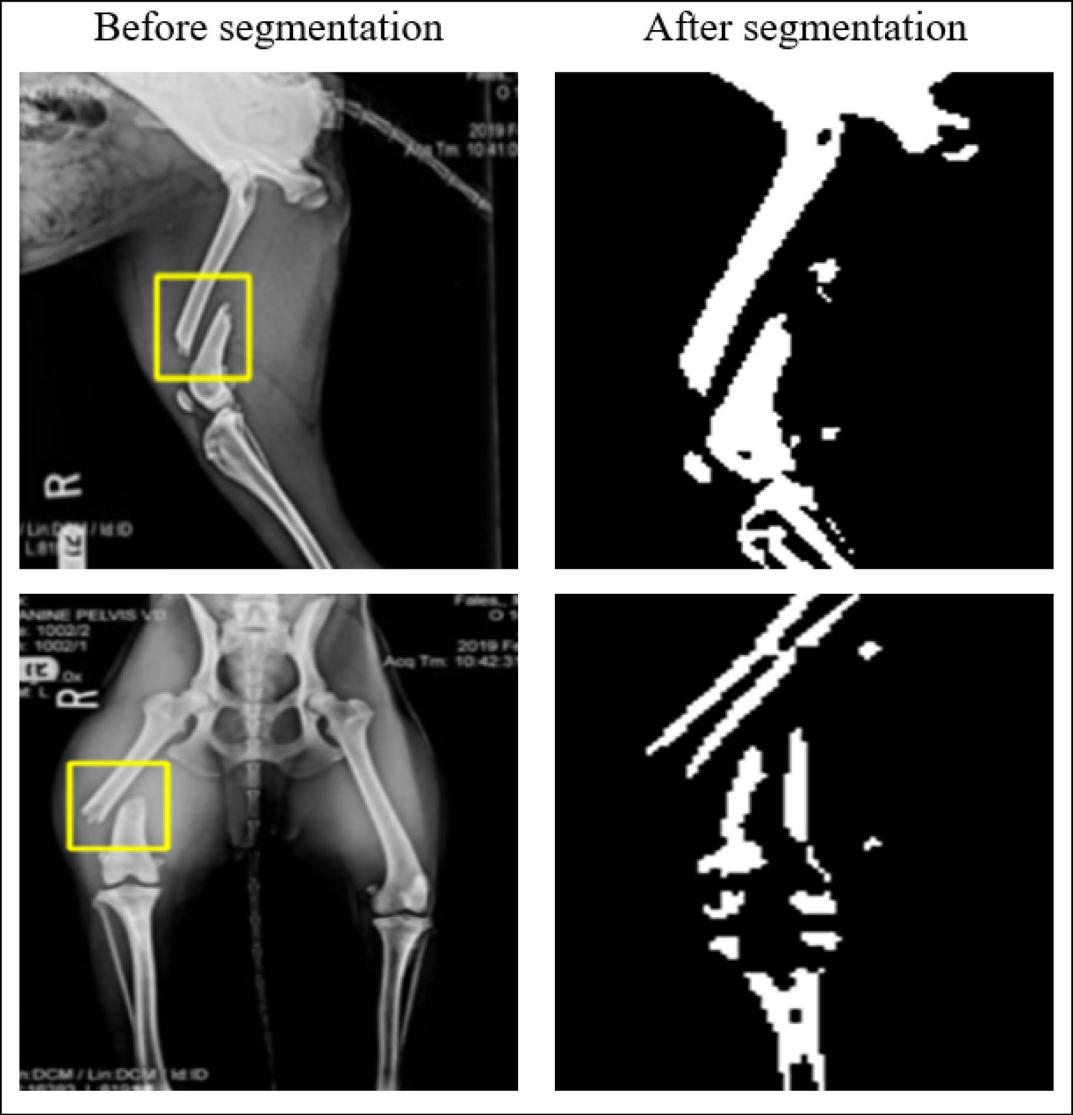
Samples of the dataset before and after segmentation.

The data segmentation has essentially proved to be a key step while maintaining the integrity of basic visual information and at the same time effectively eliminating unnecessary components. This resulted in a cleaner and more refined dataset, demonstrably more suitable for downstream tasks such as element extraction and finally for robust and reliable classification. Figure 3 visually illustrates the impact of segmentation on the dataset and clearly demonstrates the increased image quality achieved by removing irrelevant elements and improved focus on relevant visual information.

#### Data augmentation

One of the key challenges in training ML models is to ensure that the dataset is large enough to allow accurate and generalizable predictions. In our case, the initial data file was relatively small, only 29 images for a prevailing class and 15 images for an oblique class. Such a limited number of samples can reduce the ability of the model to learn robust function, leading to suboptimal performance and reduced accuracy. To solve this restriction, we used data augmentation techniques for augmented dataset extension and improve the model training process^22^.

The primary goal of enlargement of data in our methodology was to increase the number of training samples, allowing the model to learn more diverse and representative functions. By generating other variations of existing images, we focused on improving the ability of the model to generalize to invisible data and increase its overall predictive accuracy. In order to achieve this, we used the keraras Imagedatagener algorithm. This configuration used a number of transformations to create a variety of but realistic samples while maintaining key visual elements:

- Rotation: Images were randomly rotated by up to 40 degrees to simulate variations in orientation.
- Zooming: Random zooming within a range of 20% was used to simulate variations in scale and distance.
- Filling Mode: New pixels introduced during transformations were filled with a constant value to maintain consistency in the background.

**Fig. 4:**
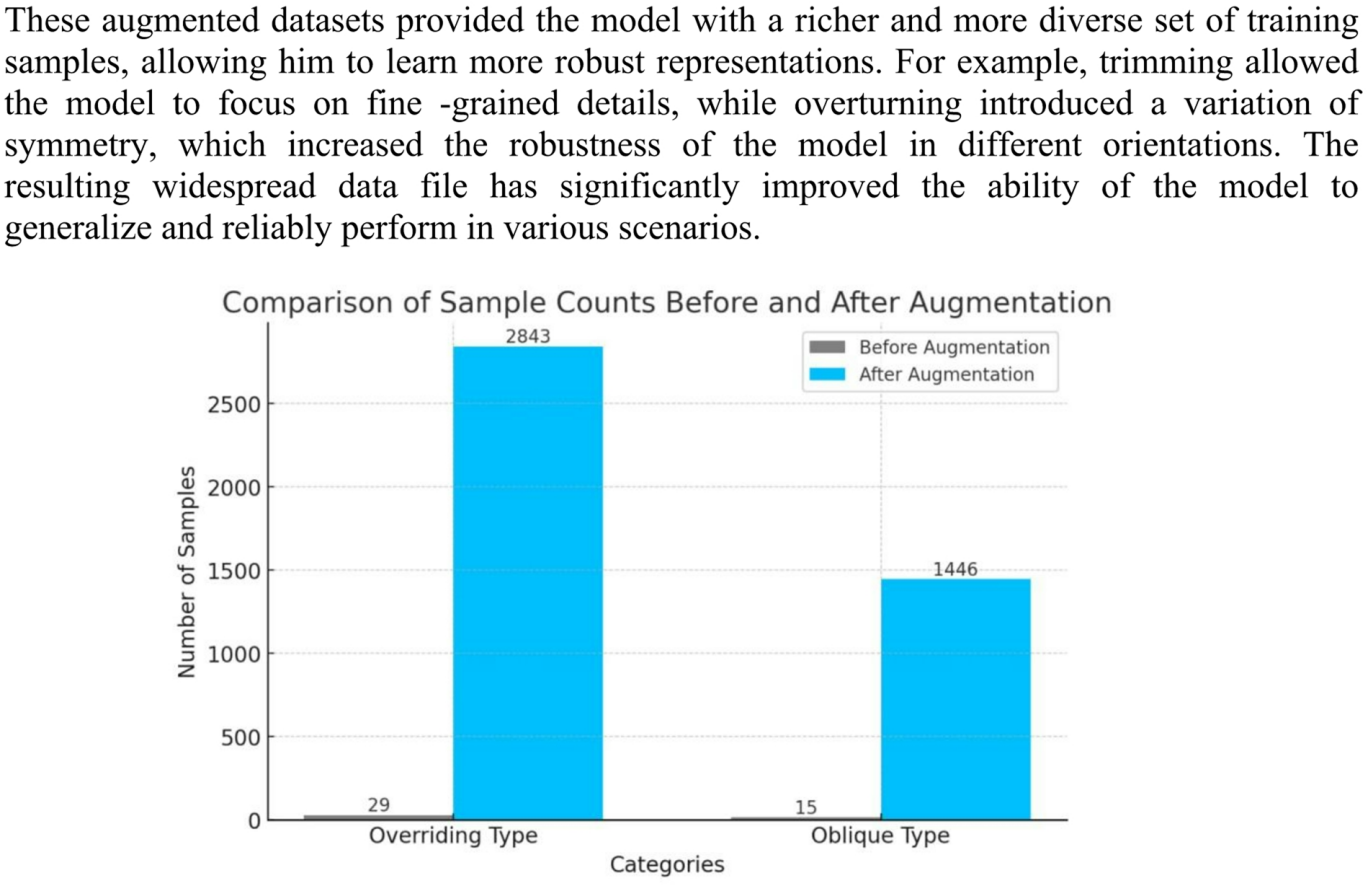
Dataset before and after augmentation.

Through this process, the dataset was significantly expanded as presented in Fig. 4:

- The Overriding class grew from 29 images to 2,843 images.
- The Oblique class increased from 15 images to 1446 images.

#### Balancing data

**Fig. 5:**
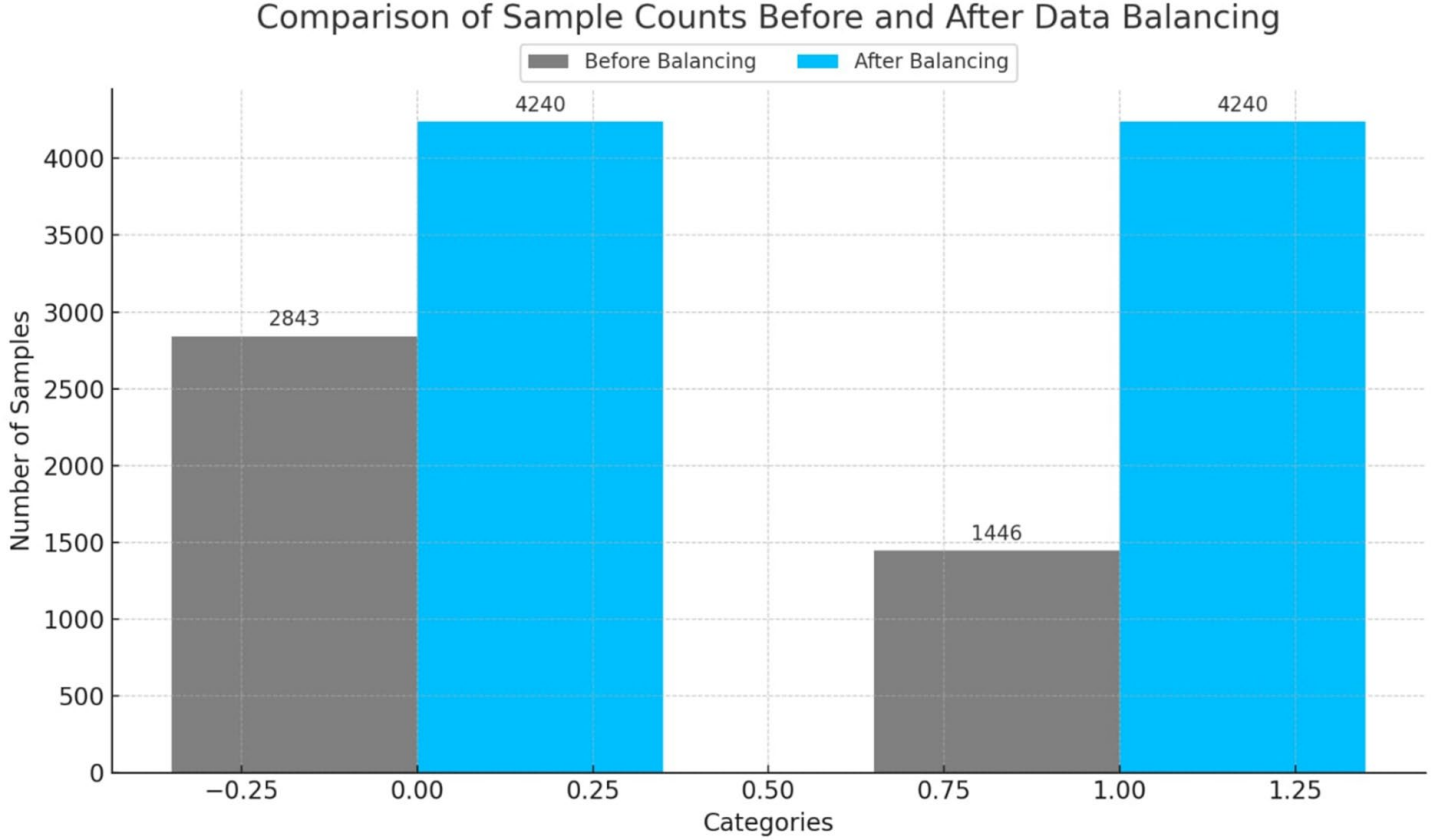
The dataset before and after balancing.

The effectiveness of any machine learning model heavily depends on the balance of its training data^23^. Figure 5 shows the distribution of sample in dog bones fracture dataset before and after use of data balance techniques. Initially, there was a significant imbalance between the two classes: the oblique class consisted of 1446 samples, while the predominant class contained 2,843 samples. Although the difference between the two classes was relatively small, even a minor imbalance can bring challenges during training, including:

- Bias Toward the Majority Class: Models trained on imbalanced datasets may prioritize the majority class, neglect the minority class and yielding suboptimal results.
- Poor Generalization: Imbalanced data can lead to overfitting, where the model fails to generalize well to unseen data, particularly for underrepresented categories.

To solve these problems and to ensure fair representation, we have implemented the strategy of data balance using data augmentation. Specifically, we used algorithm keras Imagedatagenerator. This configuration used a number of geometric and photometric transformations to generate other samples for the minority class (oblique), which ensured that both classes have the same number of samples. After balance, each class contained 4,240 samples, eliminating any differences between the two categories.

The augmentation process introduced realistic variations in the images while preserving key visual features. We applied data augmentation to the training images, including random rotation (up to 40°), shearing, and zooming (up to 20%). Newly created pixels were handled using a constant fill mode to keep the background consistent.

By equalizing the sample sizes in both classes, we have assured that the model learns equivalently from each category, hence enhancing its classification accuracy.

#### Dataset splitting

In order to ensure a robust and reliable evaluation of the model’s performance, the dataset was divided into three different subgroups: training, validation and testing^24^. The data was divided as follows:

- 80% for training: used to train the model by iterative adjustment of its parameters.
- 10% for validation: employed to tune hyperparameters and monitor the performance of the model during training, helping to prevent overfitting.
- 10% for testing: Reserved for impartial evaluation of a fully trained model, ensuring that its performance is well generalized to invisible data.

The splitting strategy balances training data requirements with independent subsets for verification and testing. The training set provides meaningful formulas, validation plays a critical role in fine nine hyperparameters, and the test set assesses model generalization to new samples.

The model was trained using 80% of the training data, ensuring a diverse set of examples for robust learning. A 10% validation set monitored model progress without risking test set integrity, ensuring representative results.

### Phase 2: feature extraction and model training

In this phase, we employed ResNet50 architecture^25^ to extraction of elements and model training. The ResNet50 is a deep convolutional neural network consisting of 50 layers that is designed to solve the problem with the disappearing gradient through residual learning. The architecture is structured into multiple phases, each of which involves convolutional layers, normalization and activation function, followed by increasing the gradient flow.

**Fig. 6:**
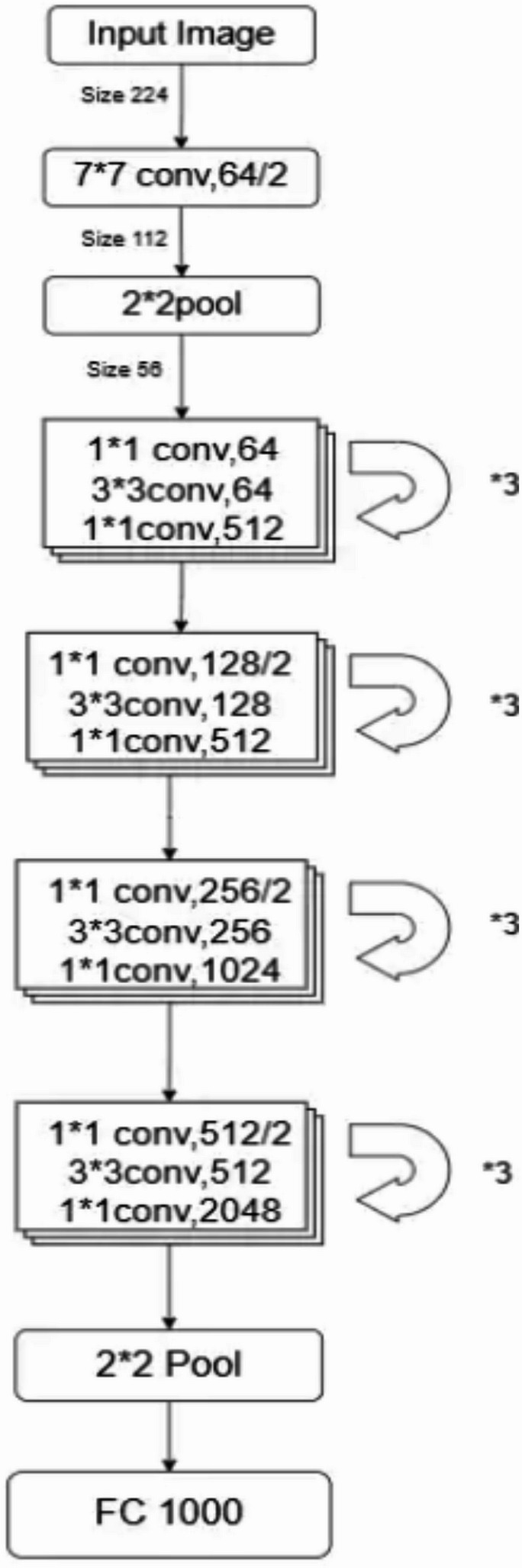
The proposed ResNet50 architecture.

The model as presented in Fig. 6 begins with an initial 7 × 7 convolutional layer with 64 filters and a stride of 2, processing the input image of size 224 × 224 and reducing it to 112 × 112. This is followed by a 2 × 2 max pooling layer, further reducing the spatial dimensions to 56 × 56.

The core of ResNet50 consists of four main residual stages, each containing multiple residual blocks. Each block comprises three convolutions:

1. A 1 × 1 convolution for reducing the dimensionality,
2. A 3 × 3 convolution for feature extraction,
3. A 1 × 1 convolution for restoring the dimensionality.

These residual blocks allow the network to retain important gradient information, mitigating the vanishing gradient issue. The stages are as follows:

- Stage 1: Three residual blocks with (64, 64, 256) filters, maintaining a feature map size of 56 × 56.
- Stage 2: Three residual blocks with (128, 128, 512) filters, reducing the spatial size to 28 × 28.
- Stage 3: Three residual blocks with (256, 256, 1024) filters, reducing the spatial size to 14 × 14.
- Stage 4: Three residual blocks with (512, 512, 2048) filters, reducing the spatial size to 7 × 7.

Following the final convolutional block, a 2 × 2 pooling layer further down samples the features before passing them to a fully connected layer with 1000 output neurons, typically used for classification tasks.

During training, the model is optimized using the Backpropagation algorithm with an adaptive optimization technique such as Adam. The normalization of the batch ensures stable learning, while the residual connection improves convergence, which makes the ResNet50 ideal architecture for deep extraction of elements in comprehensive image recognition tasks.

### Phase 3: performance evaluation

The proposed ResNet5 model is evaluated for effectiveness and efficiency after training. Performance metrics are used to assess its practicality in real-world scenarios, with accuracy being a key indicator of the model’s overall predictive performance^22^. Accuracy, as defined in Eq. (1), is the proportion of correctly predicted samples to total predictions, indicating the model’s overall predictive performance.

1 $$\:Accuracy=(TP+TN)/(TP+TN+FP+FN)$$

Precision, as defined by Eq. (2), refers to the proportion of correctly predicted positive instances for a class compared to all positive predictions made for that class.

2 $$\:Precision=TP/(TP+FP)$$

Sensitivity, or recall, is the proportion of real positive samples correctly recognized as positive, according to Eq. (3).

3 $$\:Sensitivity\vee\:Recall=TP/(TP+FN)$$

Specificity, as delineated by Eq. (4), quantifies the proportion of accurately detected negative samples.

4 $$\:Specificity=TN/(TN+FP)$$

The F1 score is calculated by weighted averaging of precision and recall, as seen in Eq. (5).

5 $$\:F1score=2\times\:\left(\right(precision\times\:recall)/(precision+recall\left)\right)$$

where FP represents false positive, and FN represents false negative.

## Result analysis and discussion

### Experimental setup

The deployment of the proposed model is developed using a personal computer with the following specifications: CPU: Intel(R) Core (TM) i5-10300 H, GPU: Nvidia GTX 1650, RAM: 8 GB, Storage: 500 GB SSD, and Operating System: 64-bit Windows 11. The code is developed using the Python programming language (version 3) with the TensorFlow framework (version 2.18.1). Additional libraries and dependencies include NumPy, Pandas, and Matplotlib.

### Performance evaluation of the proposed ResNet50 model versus other pre-trained models for the dog’s long bone fractures dataset

To evaluate the performance of the proposed ResNet50 model, parameters were carefully selected to optimize the process of learning the model and at the same time ensured generalization and robustness. During the training phase, the following parameters were set; learning level, optimizer, batch size, loss function, number of epochs, time stop criteria, input image size and data splitting ratios. The exact values ​​of these hyperparameters are summarized in Table 2.

**Table 2:** Settings of the training parameters.

Parameter	Settings
Learning rate	0.0001
Optimizer	Adam optimizer
Batch size	64
Loss function	Binary cross entropy
Number of Epochs	50
Patience in early stopping	5
Image size	224 width * 224 heights
Splitting ratio	80% train, 10% validate, 10% test

The performance of the proposed ResNet50 is shown in Table 3 and presents its excellent classification capabilities compared to other state-of-the-art DL pre-trained models. The ResNet50 achieves an exceptional accuracy of 99.76%, precision of 99.53%, recall 100% and F1-score 99.76%. These results emphasize the model’s ability to provide highly consistent and reliable classification results and overcome competitors such as EfficientNetB0 (accuracy: 99.10%) and Xception (accuracy: 99.53%). In addition to its high accuracy, the Resnet50 shows a strong balance between precision and recall, achieving a well -rounded F1- score that underlines its robustness for binary classification tasks.

**Table 3:** Comparison between the proposed ResNet50 model versus other state-of-the-art models.

Model	Accuracy	Precision	Recall	F1 score
ResNet50	0.9976	0.9953	1.0000	0.9976
VGG19	0.9917	0.9906	0.9929	0.9918
VGG16	0.9929	0.9953	0.9906	0.9929
MobileNetV2	0.9765	0.9753	0.9729	0.9764
Xception	0.9953	0.9953	0.9953	0.9953
EfficientNetB0	0.9910	0.9976	1.0000	0.9910
DenseNet121	0.9965	0.9976	0.9953	0.9965

**Fig. 7:**
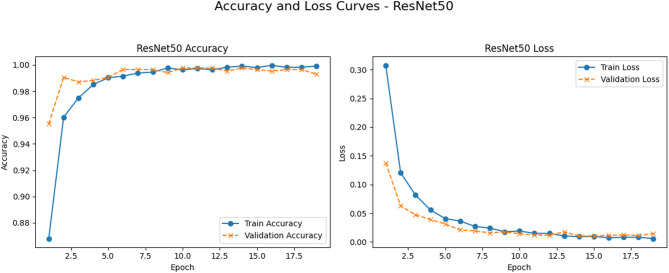
Accuracy and loss curves of the ResNet model.

Figure 7 depicts the training and validation performance of the proposed model. The loss curves exhibit a steady downward trajectory, indicating the successful learning process of the proposed model. The training curve demonstrates a continual increase in accuracy, achieving about 99%.

**Fig. 8:**
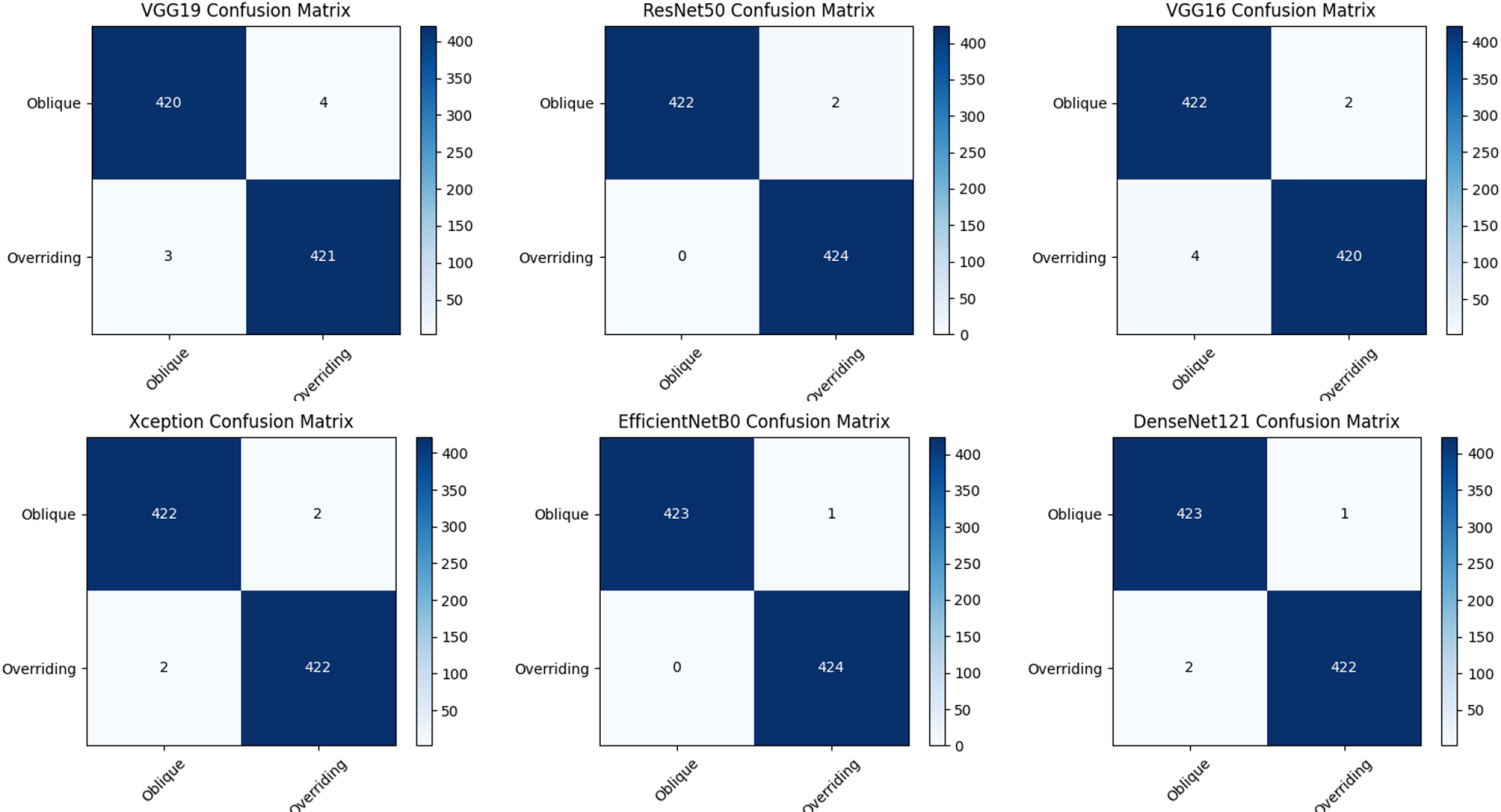
Confusion Matrix of the proposed models.

Figure 8 presents the classification performance of seven pre-trained DL models through their relevant confused matrices, with the most effective ResNet50. The ResNet50 demonstrates excellent performance by achieving the smallest false positives while maintaining a strong balance between real positives and real negatives, minimizing incorrect classification. In contrast, models such as VGG16 and VGG19, although they excel in the identification of positive cases, tend to produce a higher number of false positives, which reduces their overall accuracy.

**Fig. 9:**
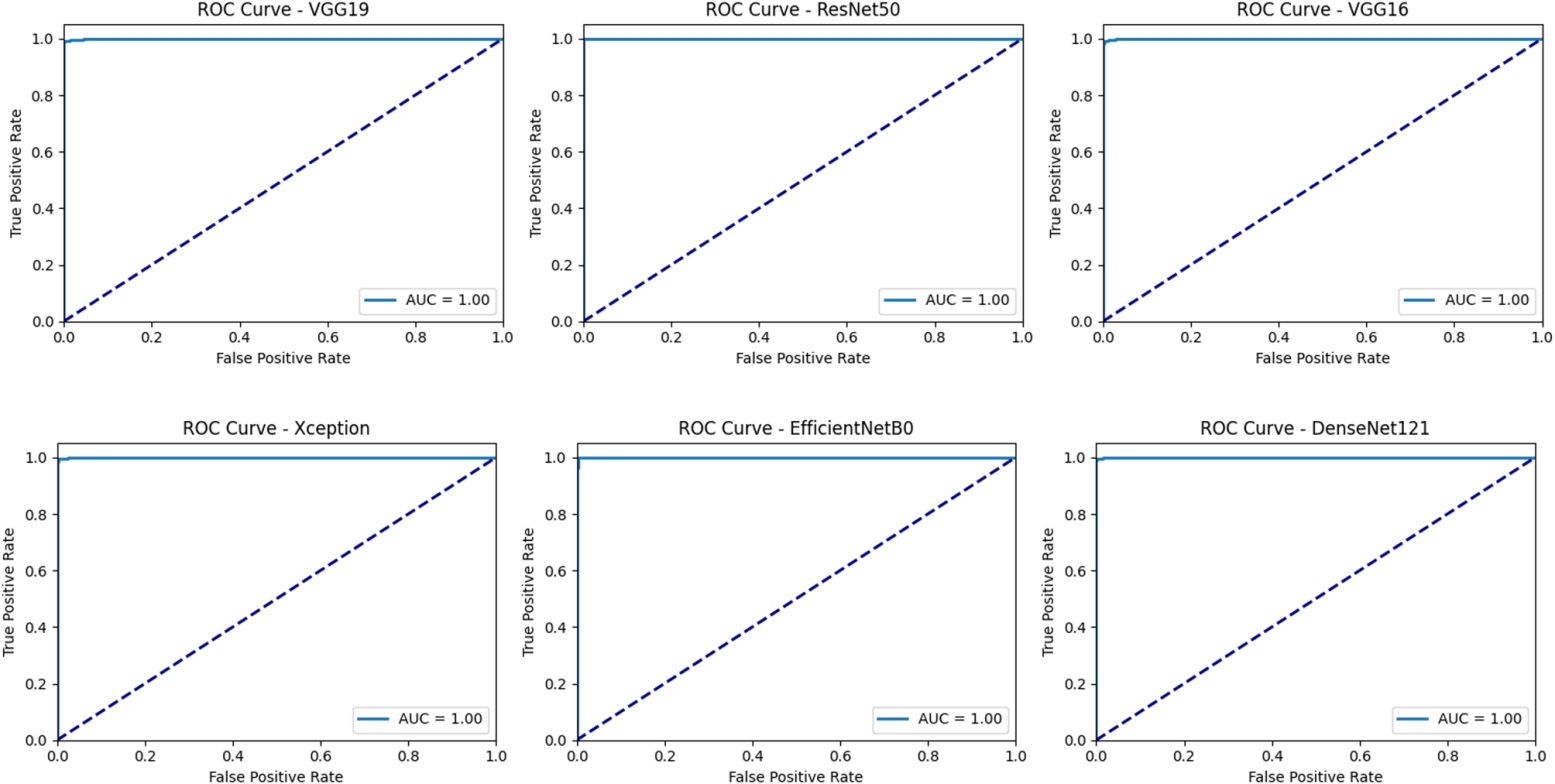
ROC curves of the ResNet50 model versus other pre-trained DL models.

Figure 9 presents the ROC (Receiver Operating Characteristic) curves^26^ and AUC (Area Under the Curve) scores^27^ of the seven pre-trained DL models, emphasize their ability to distinguish between classes. Among the evaluated architectures, the ResNet50 shows the best classification performance and robustness, reaches the highest score AUC of 1. Other models such as MobileNetV2 and Densenet121, reach a decent score of approximately 0.96 and 0.93, but show slightly greater overlapping of the class prediction. In particular, the VGG16 works the least efficiently, with a significantly lower AUC 0.72. Excellent score of AUC Model ResNet50 in combination with the results of a balanced confusion matrix fixing its location as the architecture of the highest performance for this classification task.

Based on the pairwise comparisons summarized in Table 4, all reported p-values are below 0.005, indicating that the differences in ROC–AUC between ResNet50 and each of the other evaluated architectures are statistically significant at the 0.5% level (i.e., we reject the null hypothesis of equal AUCs for each pairwise test). This supports the conclusion that ResNet50 provides significantly better discriminatory performance on the held-out test set compared with MobileNetV2, DenseNet121, and the remaining models, consistent with the clearer separation observed in the ROC curves and the more balanced confusion matrix.

**Table 4:** Pairwise ROC–AUC comparisons vs. ResNet50 (after image preprocessing).

Comparison (paired, same test set)	AUC (ResNet50)	AUC (other model)	*p*-value (paired AUC test)
ResNet50 vs. MobileNetV2	1.00	0.96	0.001
ResNet50 vs. DenseNet121	1.00	0.93	0.004
ResNet50 vs. InceptionV3	1.00	0.95	0.003
ResNet50 vs. EfficientNetB0	1.00	0.94	0.004
ResNet50 vs. Xception	1.00	0.92	0.002
ResNet50 vs. VGG16	1.00	0.72	0.001

### Computational efficiency analysis

The computational efficiency analysis is essential to verify the power of the DL model by balancing complexity and accuracy calculations and allows developers to design real-time applications and sustainable AI solutions^28^.

The computing efficiency analysis in Table 5 emphasizes the excellent performance of the proposed Resnet50 compared to other DL architectures. While the EfectiveNetB0 records the lowest MFLOPS (753.30) and the fastest inference time (0.0858s), the proposed Resnet50 follows with 802.10 MFLOPS and an inference time of 0.1004s, reaching the lowest memory consumption (98.93 MB), model size (11.67 MB) and energy consumption (11177 J). This balance of low computational complexity, high accuracy and minimal energy consumption makes the proposed Resnet50 highly efficient and suitable for deployment in resource-limited environment, overcoming larger models such as VGG19, VGG16 and XCEPCE in total computational efficiency.

**Table 5:** Computational efficiency analysis of the proposed modified ResNet50 versus other DL models.

Model	MFLOPs	Inference time (Second / image)	Memory consumption (MB)	Model size (MB)	Energy consumption (J)
VGG19	39038.39	0.1447	147.29	81.16	66,152
VGG16	30713.49	0.1088	120.84	59.92	51,503
MobileNetV2	1614.04	0.1550	247.03	98.56	28,864
Xception	16771.72	0.1536	339.58	87.65	58,729
EfficientNetB0	**753.30**	**0.0858**	119.89	18.83	15,479
DenseNet121	5701.47	0.1628	229.96	30.26	32,637
**ResNet50 (proposed)**	802.10	0.1004	**98.93**	**11.67**	**11,177**

### Ablation analysis

Table 6 presents the ablation analysis of the proposed model. Before preliminary processing, the proposed ResNet50 shows strong results with an accuracy of 0.8333 and F1-score 0.8889, which exceeds most of the other competing models. After using the pre-processing methodology, the model achieves the highest score across all metrics with an accuracy of 0.9976, the accuracy of 0.9953, the invocation of 1.0 and F1-score 0.9976, indicating almost perfect classification performance.

**Table 6:** The ablation analysis of the proposed ResNet50 model.

Model	Before image pre-processing				After image pre-processing			
	Accuracy	Precision	Recall	F1 Score	Accuracy	Precision	Recall	F1 score
VGG19	0.6667	0.6667	1.0	0.8000	0.9917	0.9906	0.9929	0.9918
VGG16	0.6667	0.6667	1.0	0.8000	0.9929	0.9953	0.9906	0.9929
MobileNetV2	0.6667	0.6667	1.0	0.8000	0.9765	0.9753	0.9729	0.9764
Xception	0.8333	0.8000	1.0	0.8889	0.9953	0.9953	0.9953	0.9953
EfficientNetB0	0.6667	1.000	0.5	0.6667	0.9910	0.9976	1.0000	0.9910
DenseNet121	0.6667	0.6667	1.0	0.8000	0.9965	0.9976	0.9953	0.9965
**ResNet50 (proposed)**	**0.8333**	**0.8000**	**1.0**	**0.8889**	**0.9976**	**0.9953**	**1.000**	**0.9976**

Table 7 provides the AUC values for ResNet50 and each comparator model together with the corresponding p-values, allowing an explicit statistical assessment of the performance differences. As shown in Table 7, all pairwise comparisons yield small p-values (≤ 0.005), indicating that ResNet50 achieves a statistically significant improvement in class discrimination compared with the competing architectures; this strengthens and supports our conclusions drawn from the ROC curves and AUC analysis.

**Table 7:** Pairwise ROC–AUC comparisons vs. ResNet50 (before image preprocessing) .

Comparison (paired, same test set)	AUC (ResNet50)	AUC (Other model)	*p*-value (paired AUC test)
ResNet50 vs. MobileNetV2	0.98	0.96	0.004
ResNet50 vs. DenseNet121	0.98	0.93	0.003
ResNet50 vs. InceptionV3	0.98	0.95	0.004
ResNet50 vs. EfficientNetB0	0.98	0.94	0.005
ResNet50 vs. Xception	0.98	0.92	0.002
ResNet50 vs. VGG16	0.98	0.72	0.001

## Conclusion and future work

This research provides an efficient deep learning model for the automated classification of oblique and overriding fractures in dogs. The promising results obtained with ResNet50, VGG16, EfectItB0, Desnet121, Xception and VGG19 are preparing a way for the development of AI -driven tools to support veterinary doctors in fracture diagnostics, leading to faster, more accurate assessment and improved patient care. Future research could focus on solving identified restrictions, increasing the interpretability of the model and transferring these promising findings into clinically relevant applications in favour of orthopaedic practice and patient results in fracture treatment.

## Data Availability

The data used in this paper will be available upon request. Contact: prof. Ashraf Sobhy Saber at email: Saberashraf_2@yahoo.com.
